# An improved auxin-inducible degron system for fission yeast

**DOI:** 10.1093/g3journal/jkab393

**Published:** 2021-11-25

**Authors:** Xiao-Ran Zhang, Lei Zhao, Fang Suo, Yadong Gao, Qingcui Wu, Xiangbing Qi, Li-Lin Du

**Affiliations:** 1 National Institute of Biological Sciences, Beijing 102206, China; 2 School of Chemical Engineering, Nanjing University of Science and Technology, Nanjing, Jiangsu 210094, China; 3 Tsinghua Institute of Multidisciplinary Biomedical Research, Tsinghua University, Beijing 102206, China

**Keywords:** auxin-inducible degron (AID), 5-adamantyl-IAA, fission yeast, *Schizosaccharomyces pombe*

## Abstract

Conditional degron technologies, which allow a protein of interest to be degraded in an inducible manner, are important tools for biological research, and are especially useful for creating conditional loss-of-function mutants of essential genes. The auxin-inducible degron (AID) technology, which utilizes plant auxin signaling components to control protein degradation in nonplant species, is a widely used small-molecular-controlled degradation method in yeasts and animals. However, the currently available AID systems still have room for further optimization. Here, we have improved the AID system for the fission yeast *Schizosaccharomyces pombe* by optimizing all three components: the AID degron, the small-molecule inducer, and the inducer-responsive F-box protein. We chose a 36-amino-acid sequence of the *Arabidopsis* IAA17 protein as the degron and employed three tandem copies of it to enhance efficiency. To minimize undesirable side effects of the inducer, we adopted a bulky analog of auxin, 5-adamantyl-IAA, and paired it with the F-box protein OsTIR1 that harbors a mutation (F74A) at the auxin-binding pocket. 5-adamantyl-IAA, when utilized with OsTIR1-F74A, is effective at concentrations thousands of times lower than auxin used in combination with wild-type OsTIR1. We tested our improved AID system on 10 essential genes and achieved inducible lethality for all of them, including ones that could not be effectively inactivated using a previously published AID system. Our improved AID system should facilitate the construction of conditional loss-of-function mutants in fission yeast.

## Introduction

Studies of gene functions, especially the functions of essential genes, are greatly assisted by the conditional inactivation of genes. Diverse strategies have been employed to achieve conditional disruption of gene functions. For model yeast species, these strategies include creating temperature-sensitive mutants (Dohmen *et al.* 1994; Kanemaki *et al.* 2003; Ben-Aroya *et al.* 2008), placing a target gene under the control of a repressible promoter (Mnaimneh *et al.* 2004), and chemically induced alteration of the localization of the gene product (Haruki *et al.* 2008). One particularly powerful strategy that has gained wide-spread use in recent years is the auxin-inducible degron (AID) technology, which adopts a protein degradation mechanism in plants for conditional depletion of proteins in nonplant organisms (Nishimura *et al.* 2009; Kanke *et al.* 2011; Nishimura and Kanemaki 2014; Zhang *et al.* 2015; Natsume *et al.* 2016; Natsume and Kanemaki 2017; Texier *et al.* 2018).

In plants, auxin, a plant hormone, promotes the degradation of auxin/indole-3-acetic acid proteins (Aux/IAAs) by acting as a molecular glue to mediate the interactions between Aux/IAAs and transport inhibitor response 1/auxin signaling F-box proteins (TIR1/AFBs), which are F-box proteins in SCF (Skp1, Cullin, and F-box protein) E3 ubiquitin ligase complexes (Tan *et al.* 2007). Aux/IAAs and TIR1/AFBs only exist in land plants (Mutte *et al.* 2018), but when an Aux/IAA protein and a TIR1/AFB protein are introduced together into yeast or animal cells, they are sufficient to recapitulate auxin-induced degradation by acting with endogenous SCF components (Nishimura *et al.* 2009). This is the mechanistic basis of the AID technology.

An AID system is composed of three components: a degron fused to the target protein, an F-box protein, and a small molecule inducer. The IAA17 protein from *Arabidopsis thaliana* has often been used as the degron (Nishimura *et al.* 2009; Kanke *et al.* 2011). The most widely used F-box proteins in AID systems are the TIR1 proteins from *A.* *thaliana* and rice (*Oryza sativa*), which are referred to as AtTIR1 and OsTIR1, respectively. OsTIR1 works better than AtTIR1 at temperatures higher than 24°C and is a preferred choice for yeasts and mammalian cells, whose optimal growth temperatures are higher than 24°C (Nishimura *et al.* 2009).

The widely used small molecule inducer in AID systems, the auxin indole-3-acetic acid (IAA) or its synthetic analog naphthalene acetic acid (NAA), is generally assumed to be inert in nonplant species. However, over 50 years ago, physiological effects of IAA and NAA on certain yeast species have been described (Yanagishima and Masuda 1965; Kamisaka *et al.* 1967). Many fungal species are now known to synthesize auxin and/or exhibit physiological responses to auxin (Fu *et al.* 2015; Chanclud and Morel 2016). In particular, the model organism *Saccharomyces cerevisiae* is able to synthesize and secrete auxin (Rao *et al.* 2010). Furthermore, in *S. cerevisiae*, IAA promotes the morphological transition to a filamentous form, retards growth, and inhibits the activity of TORC1 (Prusty *et al.* 2004; Liu *et al.* 2016; Snyder *et al.* 2019; Nicastro *et al.* 2021), and NAA exhibits a stronger TORC1 inhibition effect than IAA (Prusty *et al.* 2004; Liu *et al.* 2016; Snyder *et al.* 2019; Nicastro *et al.* 2021). In the other model yeast, the fission yeast *Schizosaccharomyces pombe*, growth inhibition was observed in the presence of high concentrations of NAA (Kanke *et al.* 2011). Thus, using IAA or NAA as an inducer in the AID systems, especially at a high concentration, may cause unintended consequences in yeasts.

The AID technology was first adopted for use in *S. pombe* by Kanke et al. (2011). In the AID system of Kanke *et al.* (named the *i*-AID system by Kanke *et al.* and hereafter referred to as the Kanke system), plant F-box proteins were fused to *S. pombe* Skp1 to increase the efficiency of target protein depletion. However, Skp1-fused AtTIR1 is toxic to *S. pombe* when expressed from a strong promoter (Kanke *et al.* 2011). Thus, in the Kanke system, a moderate-strength promoter, *Padh15*, was used to express the Skp1-fused F-box proteins. The Kanke system was tested on 15 essential genes. Out of these genes, severe growth inhibition was observed for only 3 genes and no effect was detected for 6 genes (Kanke *et al.* 2011). Combining the Kanke system with a transcription shut-off approach resulted in conditional lethality for all 15 essential genes (Kanke *et al.* 2011). It is desirable to further improve the efficiency of the AID system for *S. pombe* so that the AID system alone is sufficient to generate conditional null phenotypes for a high percentage of genes.

Here, we report an improved AID system for *S. pombe*. We show that our system is more efficient than the Kanke system and can generate conditional lethality for all essential genes tested, including ones that are refractory to the Kanke system.

## Materials and methods

### Plasmid construction

Plasmids used in this study are listed in Table  1. The C-terminal degron tagging plasmid pDB4581 was constructed by cloning a synthetic DNA fragment into a kanMX-marked pFA6a-derived C-terminal tagging plasmid backbone. The synthetic DNA fragment, which contains the sequence encoding the XTEN16 linker (SGSETPGTSESATPES) and three tandem copies of a 36-amino-acid region of IAA17 (amino acids 71-106 of IAA17, termed sAID for short AID), was synthesized by Wuxi Qinglan Biotech. When designing the DNA sequence, we preferentially chose codons favored by *S. pombe*. To reduce the chance of recombination between the three copies of sAID-coding sequences, codons were chosen so that between-copy nucleotide identities are lower than 80%.

**Table 1:** The plasmids created and used in this study

Plasmid name	Addgene ID	NBRP ID	Description	Purpose
pDB4581	171124	FYP4960	*pFA6a-3×sAID-kanMX*	3×sAID C-terminal tagging
pDB4697	171125	FYP4961	vector_*ura4*-integrating	No OsTIR1 control
pDB5050	171126	FYP4962	*Padh1-OsTIR1*_*ura4*-integrating	Expressing wild-type OsTIR1 (paired with NAA)
pDB4695	171127	FYP4963	*Padh1-OsTIR1-F74A*_*ura4*-integrating	Expressing OsTIR1-F74A (paired with 5-adamantyl-IAA)

Plasmids for integrating the OsTIR1 expression cassettes are based on the vector plasmid pDB4697. pDB4697 was constructed by cloning a 2567-bp *ura4*-gene-containing sequence from the *S. pombe* genome (chromosome III: 114,830–117,653) into the pEASY-blunt vector (TransGen Biotech) and placing it between two NotI sites. This sequence contains the 1.8-kb region deleted in *ura4-D18* and sequences flanking the 1.8-kb deleted region (about 400 bp on each side). *Padh1-OsTIR1* integrating plasmid pDB5050 was constructed by cloning the *S. pombe Padh1* promoter, OsTIR1 coding sequence codon-optimized for *S. pombe* (synthesized by Wuxi Qinglan Biotech), and the terminator of *S. cerevisiae ADH1* gene into an AvrII site about 70 bp downstream of the coding sequence of *ura4* in pDB4697. *Padh1-OsTIR1-F74A* integrating plasmid pDB4695 was constructed by mutating the codon for amino acid 74 of OsTIR1 from TTT (Phe) to GCT (Ala). Primers used for plasmid construction are listed in Supplementary Table S1. pDB4581, pDB4697, pDB5050, and pDB4695 have been deposited at Addgene (Addgene IDs 171124–171127) and the Yeast Genetic Resource Center of Japan (YGRC/NBRP) (https://yeast.nig.ac.jp, last accessed November 19, 2021) (NBRP IDs FYP4960–4963).

### Strain construction

Fission yeast strains used in this study are listed in Supplementary Table S2. Strains constructed by Kanke *et al.* were acquired from the Yeast Genetic Resource Center of Japan (YGRC/NBRP) (https://yeast.nig.ac.jp, last accessed November 19, 2021). The IAA17-tagged alleles and the F-box-protein-expressing cassettes generated by Kanke *et al.* were combined with AID components created in this study by crossing. To integrate the *Padh1-OsTIR1* cassette or the *Padh1-OsTIR1-F74A* cassette into the genome, we transformed a *ura4-D18* strain with NotI-digested pDB5050 or pDB4695. Ends-out recombination led to the integration of the cassette and converted the strain to *ura4^+^*. Integration at the *ura4* locus was verified by PCR. Endogenous C-terminal tagging of target genes with the 3×sAID degron was performed using a PCR-based tagging method. Briefly, in step 1, the degron cassette was amplified from pDB4581 using primers TAG-F and TAG-R and two homology arms were amplified from genomic DNA using the primer pair of orf_F and up_ov_R3 and the primer pair of dn_ov_F3 and dn_R, respectively. In step 2, overlap PCR using the three PCR products from step 1 as templates and orf_F and dn_R as primers generated the final PCR product used for transformation. Transformation mix was spread on YES plates, incubated at 30°C for 2 days, and then replica plated on G418-containing YES plates. Primers used for strain construction are listed in Supplementary Table S1. We have deposited 10 strains, including strains with integrated *Padh1-OsTIR1* cassette or *Padh1-OsTIR1-F74A* cassette at the Yeast Genetic Resource Center of Japan (YGRC/NBRP) (https://yeast.nig.ac.jp, last accessed November 19, 2021) (NBRP IDs FY39919–39928) (Supplementary Table S2).

### Chemical synthesis of 5-adamantyl-IAA

5-adamantyl-IAA was synthesized using a protocol modified from two previously reported procedures (Petit *et al.*2009; Sämann *et al.* 2014). First, 5-adamantyl-indole was generated through Negishi coupling of 5-iodoindole and an adamantylzinc reagent, which was prepared from the corresponding adamantylmagnesium reagent via transmetallation. 5-adamantyl-IAA was then synthesized from 5-adamantyl-indole via glyoxylation and a selective ketone reduction procedure. Full experimental details were presented in Supplementary Figure S1.

We note that 5-adamantyl-IAA has recently become available from several commercial vendors including Tokyo Chemical Industry (product number A3390).

### Culturing media

The compositions of media used in this study were as described (Forsburg and Rhind 2006). Unless otherwise specified, cells were cultured at 30°C in pombe minimal medium with glutamate (PMG) containing necessary supplements. For preparing NAA-containing media, we added into the medium 0.5 M NAA stock solution (dissolved in 1 M NaOH and stored at −20°C) and, if necessary, adjusted the pH of the medium to 6.0 using 1 M HCl. For preparing 5-adamantyl-IAA-containing media, we added into the medium 1 mM 5-adamantyl-IAA stock suspension (dissolved in 1 M NaOH and stored at −20°C; residual undissolved particles did not affect aliquoting if mixed thoroughly) and, if necessary, adjusted the pH of the medium to 6.0 using 1 M HCl. If dilution was needed before adding to the medium, the stock solution/suspension was diluted in water. The pH of the medium was not obviously affected by NaOH in the stock solution/suspension and no adjustment of pH was needed if the final dilution ratio was 1000-folds or higher, *i.e.*, if the final concentration of NAA ≤ 0.5 mM and if the final concentration of 5-adamantyl-IAA ≤ 1 μM. NAA was from Sigma (catalog number N0640).

### Spot assay

For the spot assay, cultures were grown to log phase in a liquid medium and serial fivefold dilutions of cells were spotted on plates and incubated at 30°C. After colony formation, the plates were scanned using an Epson Perfection V800 photo scanner.

### Immunoblotting

Cell lysates were prepared using a trichloroacetic acid extraction method (Ulrich and Davies 2009). Samples were separated on a 10% SDS-PAGE and immunoblotted with an anti-mini-AID antibody (MBL, Code No. M214-3) and an HRP AffiniPure goat anti-mouse IgG (H + L) secondary antibody (EarthOx, E030110-01).

### RNA-seq analysis

A log-phase culture of strain DY38751 grown in PMG liquid medium was diluted to OD600 = 0.2 and split into six equal aliquots. Into two aliquots, we added 2 mM NAA (250-fold dilution from 0.5 M NAA stock dissolved in 1 M NaOH). Into another two aliquots, we added 4 µM 5-adamantyl-IAA (250-fold dilution of 1 mM 5-adamantyl-IAA stock dissolved in 1 M NaOH). The remaining two aliquots were added with equivalent amounts of 1 M NaOH and served as controls. 1 M HCl was added to the six aliquots to adjust the pH to 6.0. These six cultures were incubated at 30°C for 6 h. Cells were then collected and RNA was isolated using the hot phenol method (Lyne *et al.* 2003). RNA-seq library preparation and Illumina sequencing were performed by Annoroad Gene Technology (Beijing, China).

Strains and plasmids are available upon request. Supplementary files available at Figshare include five supplementary figures (Supplementary Figures S1–S5) and three supplementary tables (Supplementary Tables S1–S3). Primers and strains used in this study are listed in Supplementary Tables S1 and S2, respectively. Results of the RNA-seq analysis are shown in Supplementary Table S3.

## Results

### *Padh1* promoter-expressed OsTIR1 is more effective than Skp1-fused F-box proteins used in the Kanke system

It is known that in AID systems, high expression levels of the plant F-box proteins are crucial for efficient protein depletion (Nishimura *et al.* 2009; Kanke *et al.* 2011; Nishimura and Kanemaki 2014; Zhang *et al.* 2015; Natsume *et al.* 2016; Natsume and Kanemaki 2017; Texier *et al.* 2018). In the Kanke system, the F-box protein AtTIR1 is fused with *S. pombe* Skp1 and two copies of SV40 nuclear localization signal (NLS) and is expressed from the moderate-strength *Padh15* promoter (Kanke *et al.* 2011). Because in many other species, plant F-box proteins have been successfully used without fusing to endogenous Skp1 (Nishimura *et al.* 2009; Kanke *et al.* 2011; Nishimura and Kanemaki 2014; Zhang *et al.* 2015; Natsume *et al.* 2016; Natsume and Kanemaki 2017; Texier *et al.* 2018), we envisioned that Skp1 fusion may not be necessary for *S. pombe*. Expressing the F-box proteins without Skp1 fusion may avoid toxicity and allow the F-box proteins to be expressed from a strong promoter. Thus, we decided to not fuse the F-box protein with Skp1 in our system.

The choice of fusing AtTIR1 with two copies of NLS in the Kanke system stemmed from the intention of only applying the system to degrade nuclear proteins. When AtTIR1 is concentrated inside the nucleus by the NLSs, it may no longer be effective at degrading cytoplasmic proteins. We aimed to develop a system that can be used for degrading both nuclear and cytoplasmic proteins. Because F-box proteins are relatively small (<70 kDa), they do not need an NLS to enter the nucleus and in the absence of NLS-dependent active transport into the nucleus, they can still passively diffuse through nuclear pores on the timescale of minutes (Timney *et al.* 2016). Therefore, we chose to not add an NLS to the F-box protein in our system.

**Figure 1:**
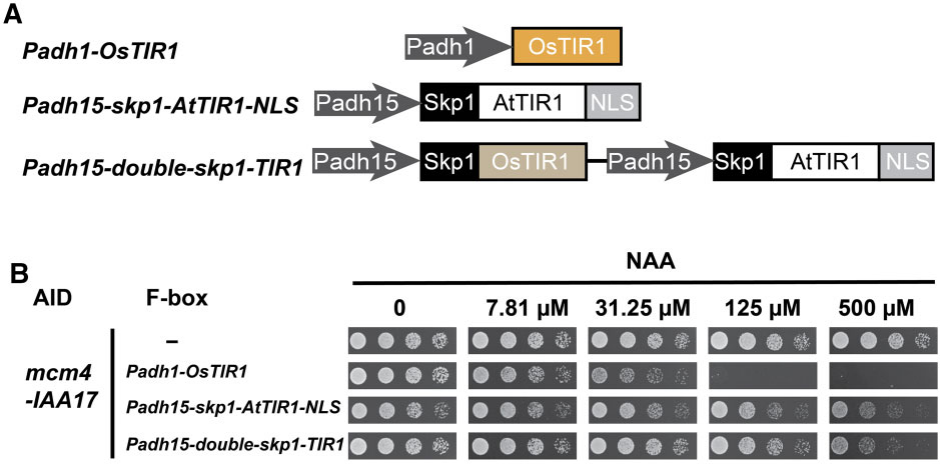
*Padh1* promoter-expressed OsTIR1 is more effective than F-box proteins used in the Kanke system. (A) Schematic of the genome-integrated cassettes expressing F-box proteins (not drawn to scale). The *Padh1-OsTIR1* cassette expressing codon-optimized OsTIR1 from the strong *Padh1* promoter was designed and constructed in this study. The *Padh15-skp1-AtTIR1-NLS* cassette and the *Padh15-double-skp1-TIR1* cassette were developed by Kanke *et al.* (2011). (B) Spot assay showed that growth inhibition occurred at a lower concentration of NAA when the *mcm4-IAA17* allele was combined with the *Padh1-OsTIR1* cassette than when combined with the *Padh15-skp1-AtTIR1-NLS* cassette or the *Padh15-double-skp1-TIR1* cassette.

An OsTIR1 coding sequence codon-optimized for *S. pombe* was obtained by gene synthesis and placed under the control of the strong *Padh1* promoter (Figure  1A). The OsTIR1-expressing cassette (hereafter referred to as *Padh1-OsTIR1*) was integrated at the *ura4* locus by ends-out recombination using the plasmid pDB5050 (Table  1 and Supplementary Figure S2A). Codon-optimized OsTIR1 expressed from the strong *Padh1* promoter showed no obvious toxicity in the absence of auxin and the AID degron (Supplementary Figure S2B).

To test the effectiveness of the *Padh1* promoter-expressed OsTIR1 in an AID system, we targeted the essential gene *mcm4*, which encodes a nuclear protein required for DNA replication. For essential genes, effective disruption of gene function manifests as growth retardation. We used the degron-tagged *mcm4* allele generated by Kanke *et al.*, *mcm4-IAA17*, in which the full-length IAA17 was used as the degron. Kanke *et al.* previously developed two versions of integrated F-box-protein-expressing cassettes, *Padh15-skp1-AtTIR1-NLS* and *Padh15-double-skp1-TIR1* (Figure  1A). Both versions, when combined with the *mcm4-IAA17* allele, resulted in obvious but incomplete growth inhibition at 30°C when the inducer NAA was added at the concentration of 500 µM, which is the concentration previously used by Kanke *et al.* (Figure  1B). In contrast, when our *Padh1-OsTIR1* cassette was combined with the *mcm4-IAA17* allele, complete growth inhibition was observed at 125 µM of NAA (Figure  1B). Because *Padh1-OsTIR1* resulted in a stronger growth inhibition at a fourfold lower concentration of inducer, we concluded that *Padh1-OsTIR1* is more effective than the F-box-protein-expressing cassettes of the Kanke system for disrupting the function of *mcm4*.

To determine whether the higher effectiveness of the *Padh1-OsTIR1* cassette holds true in general, we targeted two other essential genes *cdc45* and *orc2*, both of which encode nuclear proteins. Using the *cdc45-IAA17* and *orc2-IAA17* alleles generated by Kanke *et al.*, we found that for both genes, *Padh1-OsTIR1* was more potent than the F-box-protein-expressing cassettes developed by Kanke *et al.* (Supplementary Figure S3). Together, these results indicate that *Padh1-OsTIR1* is an improvement of the AID system for *S. pombe*.

### 3×sAID is a more effective degron than the full-length IAA17

**Figure 2:**
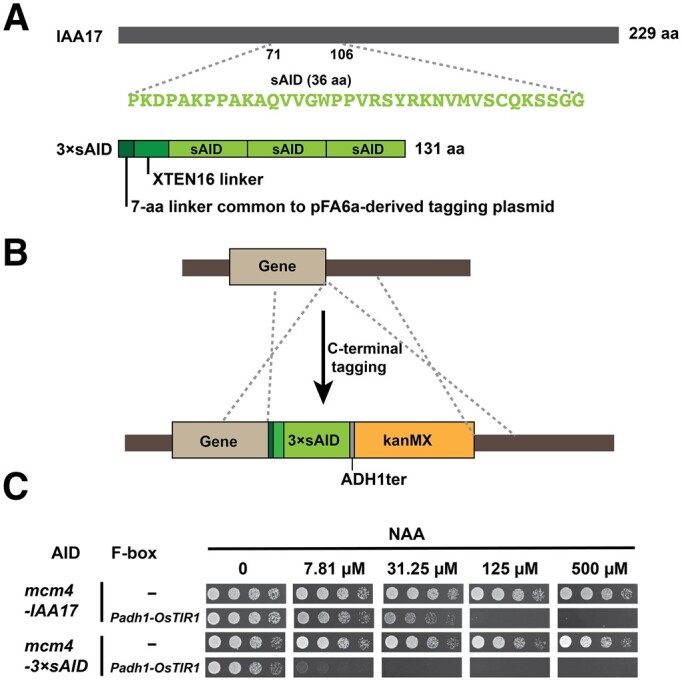
3×sAID is a more effective degron than the full-length IAA17. (A) Schematic of the 3×sAID degron in the tagging plasmid pDB4581. (B) Schematic of endogenous C-terminal tagging of a gene with the 3×sAID degron in the plasmid pDB4581 (not drawn to scale). *ADH1ter* and *kanMX* are the terminator of *S. cerevisiae ADH1* gene and the G418-resistance selection marker, respectively. (C) Spot assay showed that growth inhibition occurred at a lower concentration of NAA when the *Padh1-OsTIR1* cassette was combined with *mcm4-3×sAID* than when combined with *mcm4-IAA17*.

In the Kanke system, the full-length IAA17 is used as the degron (Kanke *et al.* 2011). To reduce the size of the degron and thereby minimize the possibility of the fused degron interfering with the function of the target protein, we selected a 36-amino-acid sequence from the IAA17 protein (amino acids 71–106 of IAA17, hereafter referred to as sAID, for short AID) based on sequence conservation and known structure-function relationship of Aux/IAA proteins (Figure  2A). sAID is shorter than previously reported small-size AID degrons derived from IAA17, including AID*, AID^47^, and mini-AID (Kubota *et al.* 2013; Morawska and Ulrich 2013; Brosh *et al.* 2016; Natsume *et al.* 2016; Yesbolatova *et al.* 2019) (Supplementary Figure S4A). Based on a previous report showing that three copies of mini-AID worked better than a single copy of mini-AID (Kubota *et al.* 2013), we used three tandem copies of the sAID sequence as the degron (hereafter referred to as 3×sAID) (Figure  2A). To avoid recombination between the three copies, we introduced synonymous variations in the three copies, so that between-copy nucleotide identities are lower than 80% (Supplementary Figure S4B).

We constructed a C-terminal 3×sAID tagging plasmid (pDB4581) (Table  1 and Figure  2B), which is based on the pFA6a-derived tagging plasmid series (Bahler *et al.* 1998; Longtine *et al.* 1998). In this plasmid, upstream of the 3×sAID degron, we added a 16-amino-acid XTEN16 linker (SGSETPGTSESATPES), which was initially developed by David Liu’s lab for linking Cas9 and FokI and has been adopted by Jonathan Weissman’s lab as a linker between an AID tag and the target protein (Guilinger *et al.* 2014; Costa *et al.* 2018). Like other pFA6a-derived C-terminal tagging plasmids, when using pDB4581 as a template for PCR-based C-terminal tagging, a 7-amino-acid linker (RIPGLIN) is present immediately downstream of the target protein. The total length of the amino acid sequence added to the target protein, including the 3×sAID degron and the linkers, is only 131 amino acids, substantially shorter than the length of full-length IAA17 (229 amino acids).

To test whether the 3×sAID degron is more effective than the full-length IAA17 used in the Kanke system, we endogenously tagged Mcm4 with the 3×sAID degron. When *mcm4-3×sAID* was combined with *Padh1-OsTIR1*, nearly complete growth inhibition was observed at 7.81 µM of NAA (Figure  2C). Because *mcm4-IAA17* in combination with *Padh1-OsTIR1* did not show as severe a growth phenotype at a fourfold higher concentration of NAA (31.25 µM) (Figure  2C), we concluded that 3×sAID is a more effective degron than the full-length IAA17 for targeting *mcm4*.

We tested the new degron on two additional essential genes *pol1* and *cdc20*. In combination with *Padh1-OsTIR1*, *pol1-IAA17* allele previously constructed by Kanke *et al.* exhibited a moderate growth inhibition at 500 µM of NAA, whereas *pol1-3*×*sAID* allele exhibited a nearly complete growth inhibition at 31.25 µM of NAA (Supplementary Figure S5A). For *cdc20*, in combination with *Padh1-OsTIR1*, the *cdc20-IAA17* allele previously constructed by Kanke *et al.* showed no obvious growth phenotype at 500 µM of NAA, whereas the same concentration of NAA completely inhibited the growth of *cdc20-3*×*sAID* strain (Supplementary Figure S5B). Together, these results indicate that 3×sAID is superior to the full-length IAA as a degron in AID systems.

### 5-adamantyl-IAA, when paired with OsTIR1-F74A, is a highly effective inducer

**Figure 3:**
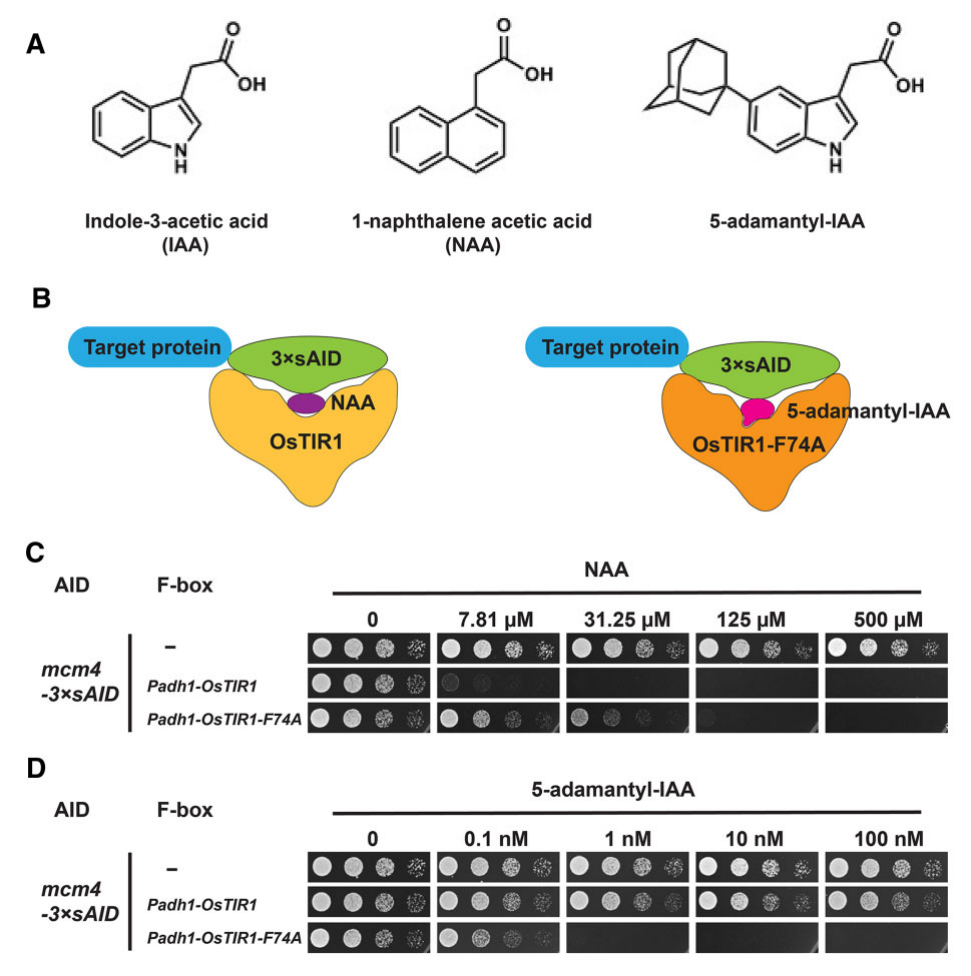
5-adamantyl-IAA is a highly effective inducer when paired with OsTIR1-F74A. (A) The structures of IAA, NAA, and 5-adamantyl-IAA. (B) Schematic showing that NAA preferentially binds OsTIR1, whereas 5-adamantyl-IAA preferentially binds OsTIR1-F74A. (C) Spot assay showed that *Padh1-OsTIR1-F74A* was less effective than *Padh1-OsTIR1* when using NAA as the inducer. (D) Spot assay showed that *Padh1-OsTIR1-F74A* but not *Padh1-OsTIR1* was effective when using 5-adamantyl-IAA as the inducer. Spot assay was performed using PMG plates incubated at 30°C.

Because auxins including IAA and NAA are known to have physiological effects on yeasts, such as growth impediment and perturbation of TORC1, we decided to adopt a synthetic auxin analog, 5-adamantyl-IAA, as the inducer for the AID system. Compared to IAA, 5-adamantyl-IAA bears an extra bulky adamantane group (Figure  3A), and thus is unlikely to cause the same physiological effects as auxins cause. Furthermore, 5-adamantyl-IAA can induce an interaction between the F79A mutant form of AtTIR1, which has an enlarged auxin-binding pocket, and Aux/IAA proteins at an extremely low concentration of 10 pM (Torii *et al.* 2018; Uchida *et al.* 2018; Yamada *et al.* 2018), suggesting the possibility of using 5-adamantyl-IAA as an AID inducer at much lower concentrations than auxins. In OsTIR1, the F74A mutation is equivalent to the F79A mutation in AtTIR1. We introduced the F74A mutation into codon-optimized OsTIR1 of the *Padh1-OsTIR1* cassette and generated a plasmid for integrating the *Padh1-OsTIR1-F74A* cassette at the *ura4* locus through ends-out recombination (pDB4695) (Figure  3B and Table  1). Like OsTIR1 expressed from the *Padh1* promoter, OsTIR1-F74A expressed from the *Padh1* promoter did not cause obvious growth inhibition in the absence of auxin and the AID degron (Supplementary Figure S2B).

**Figure 4:**
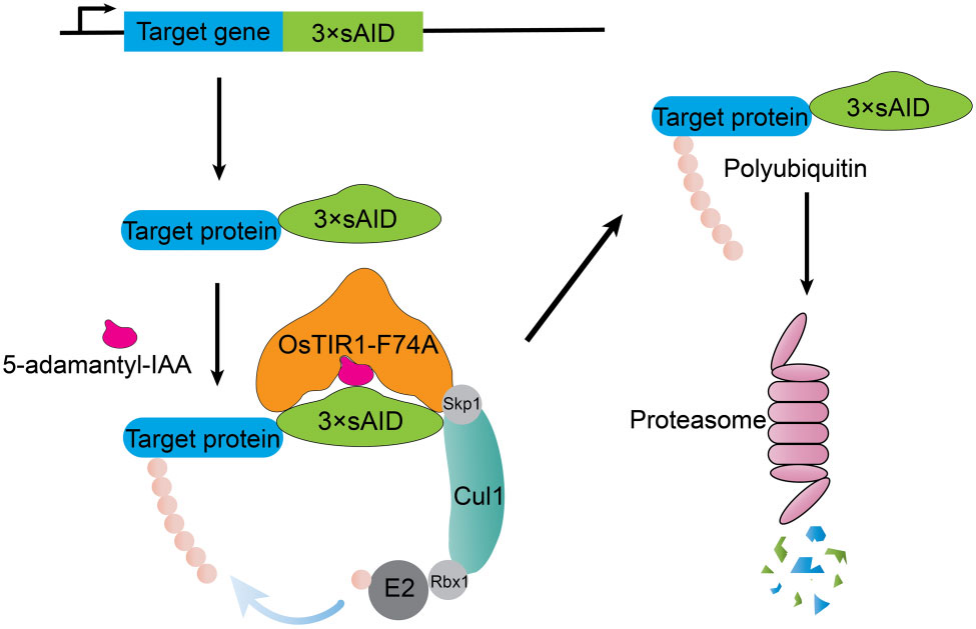
The improved AID system for *S. pombe*. Schematic of the improved AID system. OsTIR1-F74A can form a functional E3 ligase complex with endogenous SCF components. 5-adamantyl-IAA acts as a molecular glue to promote the interaction between OsTIR1-F74A and 3×sAID, resulting in the degradation of the 3×sAID fused target protein, likely in a ubiquitination- and proteasome-dependent manner.

We compared the abilities of *Padh1-OsTIR1* and *Padh1-OsTIR1-F74A* to generate growth inhibitory effects on cells harboring the *mcm4-3*×*sAID* allele in response to the two different inducers NAA and 5-adamantyl-IAA (Figure  3, C and D). As expected, *Padh1-OsTIR1-F74A* was less effective than *Padh1-OsTIR1* when using NAA as the inducer (Figure  3C), presumably because the F74A mutation makes NAA not fit well in the auxin-binding pocket. When using 5-adamantyl-IAA as the inducer, we found that, remarkably, 1 nM of 5-adamantyl-IAA was enough to completely inhibit the growth of cells containing the *Padh1-OsTIR1-F74A* cassette (Figure  3D). In contrast, hardly any growth inhibitory effect was observed at 100 nM of 5-adamantyl-IAA for cells containing the *Padh1-OsTIR1* cassette, consistent with the expectation that the bulky adamantane group of 5-adamantyl-IAA makes it unable to fit into the auxin-binding pocket of wild-type OsTIR1 (Figure  3D). The fact that 1 nM of 5-adamantyl-IAA in combination with *Padh1-OsTIR1-F74A* generated a stronger growth inhibition than 7.81 µM of NAA in combination with *Padh1-OsTIR1* (comparing the second row in Figure  3C and the third row in Figure  3D) suggests that 5-adamantyl-IAA is a highly effective inducer when paired with OsTIR1-F74A and can be used at concentrations thousands of times lower than NAA used in combination with wild-type OsTIR1. We concluded that the combination of the 3×sAID degron, the *Padh1-OsTIR1-F74A* cassette, and 5-adamantyl-IAA as the inducer constitute an improved AID system for *S. pombe* (Figure  4).

### Our improved AID system is effective on genes refractory to the Kanke system

Kanke et al. (2011) has tested their AID system on 15 essential genes, all of which encode nuclear proteins, and found that for only three of these 15 genes, severe growth phenotype can be obtained. We applied our improved AID system on eight genes previously tested by Kanke *et al.* (Table  2), including two genes (*mcm4* and *orc2*) that exhibited severe growth defect using the Kanke system, two genes (*cdc45* and *orc6*) that exhibited moderate growth defect using the Kanke system, and four genes (*pol1*, *cdc20*, *mcm10*, and *ssl3*) that exhibited no growth phenotype using the Kanke system. The improved AID system was able to generate induced lethality (complete growth inhibition on a medium containing 5-adamantyl-IAA) for all eight genes encoding nuclear proteins. Consistent with the results of Kanke *et al.* higher concentrations of 5-adamantyl-IAA were required to generate induced lethality for the genes that exhibited weaker phenotype in the Kanke system (Table  2). *cdc20*, *mcm10*, and *ssl3* required the highest concentration of 5-adamantyl-IAA (100 nM) for complete growth inhibition. We concluded that our improved AID system permits inactivation of genes refractory to the Kanke system and 100 nM of 5-adamantyl-IAA should be sufficient for generating a strong inactivating effect for most target genes.

**Table 2:** Our improved AID system is effective on genes that are refractory to the Kanke system

Essential gene	Results obtained using the Kanke system (according to Kanke *et al.*)	Results obtained using our improved AID system (the concentration of 5-adamantyl-IAA that resulted in complete growth inhibition)^a^
*mcm4*	Severe growth defect	Complete growth inhibition at 1 nM
*orc2*	Severe growth defect	Complete growth inhibition at 10 nM
*cdc45*	Slow growth	Complete growth inhibition at 10 nM
*orc6*	Slow growth	Complete growth inhibition at 10 nM
*pol1*	No effect	Complete growth inhibition at 10 nM
*cdc20*	No effect	Complete growth inhibition at 100 nM
*mcm10*	No effect	Complete growth inhibition at 100 nM
*ssl3*	No effect	Complete growth inhibition at 100 nM

a Growth phenotype was examined by spot assays using PMG plates containing 4 different concentrations of 5-adamantyl-IAA (0.1, 1, 10, and 100 nM) at 30°C.

### The improved AID system can be used for inactivating genes encoding cytoplasmic proteins

**Figure 5:**
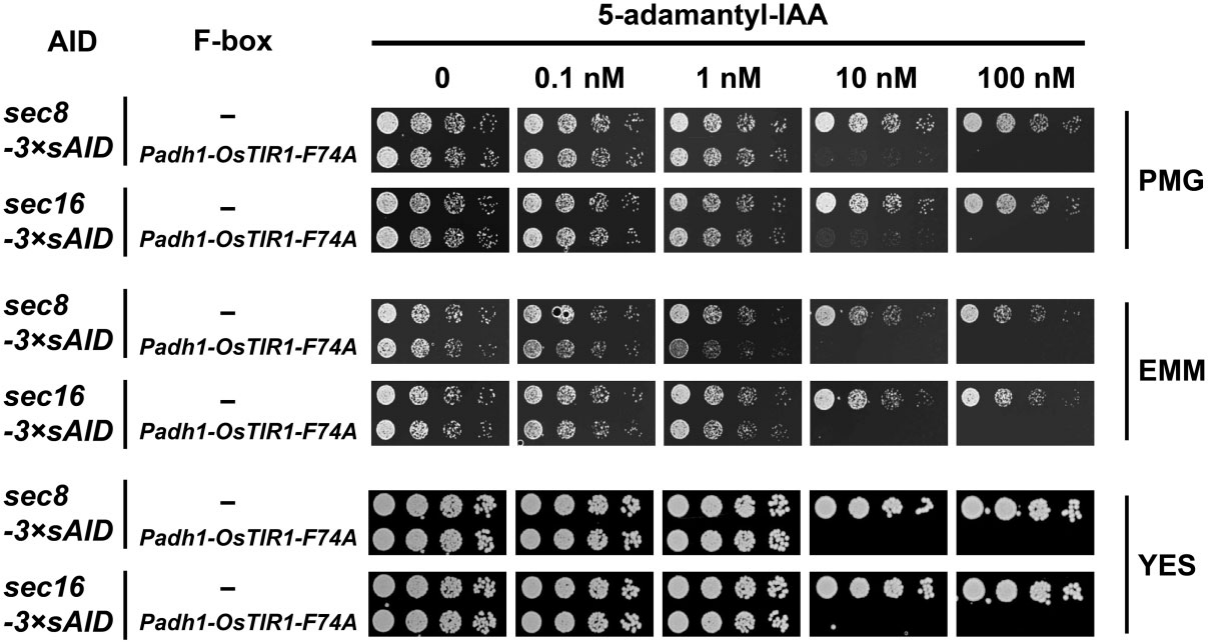
The improved AID system is effective on cytoplasmic proteins. Spot assay showed that complete growth inhibition was achieved when using the improved AID system to disrupt the functions of two essential genes encoding cytoplasmic proteins. Spot assays were performed on PMG, EMM, YES plates as indicated.

Kanke et al. (2011) has only tested their system on genes encoding nuclear proteins. In our system, we intentionally opted to not add an NLS to the F-box protein OsTIR1 so that it can target both nuclear and cytoplasmic proteins. To test whether the improved AID system is effective in disrupting the functions of genes encoding cytoplasmic proteins, we endogenously tagged two cytoplasmic proteins, Sec8 and Sec16, with 3×sAID at their C-termini. Both Sec8 and Sec16 are essential for growth. Complete growth inhibition was observed with 100 nM of 5-adamantyl-IAA for both *sec8*-*3×sAID* and *sec16*-*3×sAID* in PMG, EMM, and YES media (Figure  5). These results suggest that our improved AID system can efficiently disrupt the functions of cytoplasmic proteins in *S. pombe*, and the type of medium does not strongly affect the efficiency of the system.

### Protein depletion driven by the improved AID system

**Figure 6:**
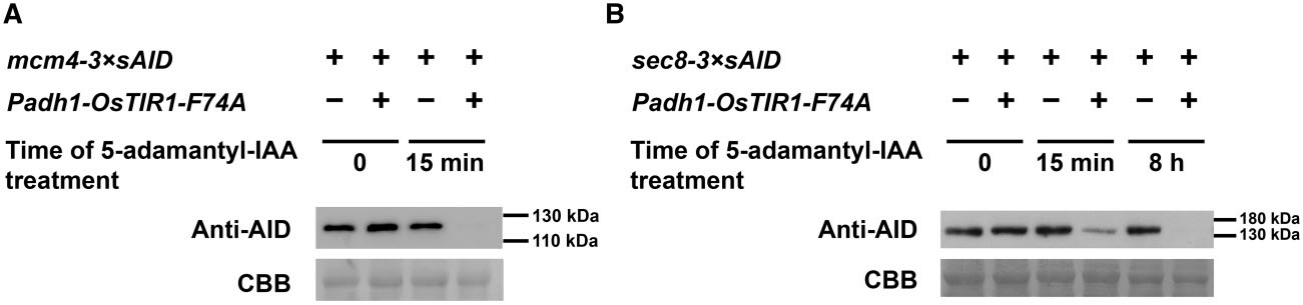
Complete or near-complete protein depletion can be achieved using the improved AID system. Two *mcm4-3×sAID* strains (A) or two *sec8-3×sAID* strains (B), one carrying *Padh1-OsTIR1-F74A* and one not, were cultured at 30°C in PMG media. The culture of each strain was split into two subcultures; one subculture was left untreated while 100 nM of 5-adamantyl-IAA was added to the other culture. After incubation for the indicated times, cells were collected and lysed. Proteins in whole-cell extracts were separated on a 10% SDS-PAGE gel and analyzed by immunoblotting with an anti-mini-AID monoclonal antibody (MBL M214-3). Coomassie brilliant blue staining of PVDF membrane was used as loading control.

To directly assess the extent of protein depletion driven by our improved AID system, we performed immunoblotting analysis (Figure  6). Mcm4-3×sAID was depleted to below-detection level after treating the cells with 5-adamantyl-IAA for 15 min (Figure  6A). Sec8-3×sAID was depleted substantially after 15 min treatment and was depleted to below-detection level after 8 h treatment (Figure  6B). These data indicate that complete or near-complete protein depletion can be achieved using the improved AID system.

### NAA but not 5-adamantyl-IAA perturbs the transcriptome

**Figure 7:**
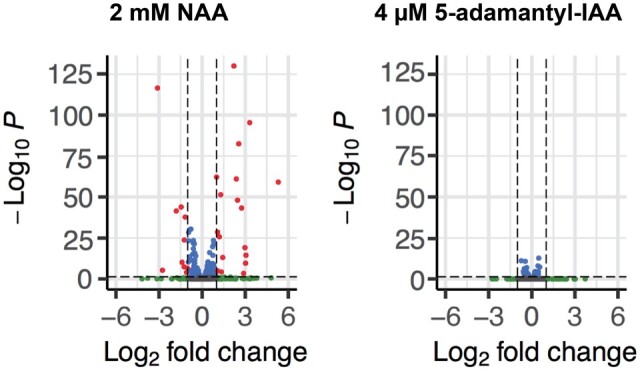
Two millimolar of NAA but not 4 μM of 5-adamantyl-IAA perturbed the transcriptome. Changes in the transcriptome are visualized using volcano plots. Left, NAA-treated samples are compared with control samples; right, 5-adamantyl-IAA-treated samples are compared with control samples. The *x*-axis shows the Log2 (fold change) in transcript levels between treated and control samples. The two vertical dashed lines denote Log2 (fold change)  = −1 and 1, respectively. The *y*-axis shows the −Log10 (adjusted *P*-value). The horizontal dashed line denotes adjusted *P*-value = 0.05. Genes with fold changes > 2 but adjusted *P*-values > 0.05 are shown in green, genes with adjusted *P*-values < 0.05 but fold changes < 2 are shown in blue, and genes with fold changes > 2 and adjusted *P*-values < 0.05 are shown in red. The smallest adjusted *P*-value for NAA treatment, that of the gene SPCC569.05c, is 5.5654E-197. For better visualization, we arbitrarily set the adjusted *P*-value of SPCC569.05c to 1E-130. For genes that were given NA values for adjusted *P*-values by DESeq2, we set their adjusted *P*-values to 1.

To experimentally assess whether NAA and 5-adamantyl-IAA may cause undesirable side effects in *S. pombe*, we performed RNA-seq analysis on *S. pombe* cells containing neither an AID degron nor a TIR1 protein. We treated cells with either 2 mM of NAA or 4 μM of 5-adamantyl-IAA for 6 h. Two millimolar of NAA is four times the concentration recommended by Kanke et al. (2011) for the Kanke system; 4 μM of 5-adamantyl-IAA is 40 times the maximal concentration we used with our improved AID system. In our hands, neither 2 mM of NAA nor 4 μM of 5-adamantyl-IAA caused an obvious growth phenotype (our unpublished observations). Using fold changes > 2 and adjusted *P*-values < 0.05 as cutoffs for differentially expressed genes, we found that 18 genes were up-regulated and 10 genes were down-regulated upon NAA treatment (Figure  7 and Supplementary Table S3). In contrast, using the same cutoffs, no differentially expressed genes were found in cells treated with 5-adamantyl-IAA (Figure  7 and Supplementary Table S3). These results indicate that a high concentration of NAA can alter the transcriptome of *S. pombe*, whereas 5-adamantyl-IAA at a concentration far higher than those used in our improved AID system had no obvious effect on the transcriptome.

## Discussion

Here, we report an improved AID system that permits efficient disruption of gene function in fission yeast. In this system, we combine an improved degron composed of three tandem copies of a 36-amino-acid sequence from IAA17, codon-optimized OsTIR1-F74A expressed from a strong promoter, and 5-adamantyl-IAA as an inducer to minimize side effects. This system is effective in disrupting the functions of 10 essential genes, including genes that are refractory to a previously reported system.

During the preparation of our manuscript, another study on improving the AID system for *S. pombe* was published (Watson *et al.* 2021). Similar to our findings, Watson *et al.* also showed that OsTIR1 not fused with Skp1 can be expressed from a strong promoter without causing toxicity and is more effective in inactivating *mcm4* than the *Padh15-skp1-AtTIR1-NLS* cassette of the Kanke system. Like the Kanke system, Watson *et al.* aimed to use their system for depleting nuclear proteins and therefore added an NLS to OsTIR1. They showed that two nuclear proteins, Mcm4 and Rad52, were effectively depleted. It is likely that using an NLS to target OsTIR1 to the nucleus can lower the efficiency of depleting cytoplasmic proteins. In our system, we intentionally chose to not add an NLS to OsTIR1 to allow broad applicability. Indeed, genes encoding both nuclear and cytoplasmic proteins can be effectively inactivated using our improved system.

We showed in this study that, when paired with OsTIR1-F74A, 5-adamantyl-IAA is a highly effective inducer for the AID system and can minimize side effects on the transcriptome. Several recently published studies have also demonstrated that a bulky auxin analog, when paired with a mutant form of OsTIR1 with an enlarged auxin-binding pocket, acts as an effective AID inducer at much lower concentrations than auxin (Nishimura *et al.* 2020; Yesbolatova *et al.* 2020; Watson *et al.* 2021). When we initiated this study in 2018, 5-adamantyl-IAA was not commercially available and therefore we obtained it by chemical synthesis. Recently, it has become available from several commercial vendors. Because 5-adamantyl-IAA can be used at very low concentrations, it is a highly cost-effective inducer. For example, the amount of 5-adamantyl-IAA needed to prepare 1 l of medium containing 100 nM of 5-adamantyl-IAA costs less than half a US dollar.

In mammalian cell lines, OsTIR1-based AID systems have been reported to cause the degradation of degron-fused target proteins and generate loss-of-function phenotypes in the absence of the auxin inducer (Natsume *et al.* 2016; Li *et al.* 2019; Sathyan *et al.* 2019; Yesbolatova *et al.* 2019). In budding yeast, auxin-independent depletion of AID-tagged proteins was also observed when OsTIR1 was expressed to a high level (Mendoza-Ochoa *et al.* 2019). This undesirable phenomenon, called “basal degradation” or “leaky degradation,” has been tackled by a number of approaches, including placing OsTIR1 under the control of a repressible promoter (Natsume *et al.* 2016; Mendoza-Ochoa *et al.* 2019), adding a small molecule antagonist of OsTIR1 (Yesbolatova *et al.* 2019), switching to a different F-box protein (Li *et al.* 2019), expressing another plant auxin signaling protein ARF (Sathyan *et al.* 2019), and using the OsTIR1-F74G mutant (Yesbolatova *et al.* 2020). It was reported that in budding yeast, OsTIR1-F74A, when expressed from the *GAL1-10* promoter, can cause inducer-independent loss-of-function phenotypes (Yesbolatova *et al.* 2020). In fission yeast, we have not noticed any obvious inducer-independent phenotypes using either wild-type OsTIR1 or OsTIR1-F74A expressed from the strong *Padh1* promoter (spot assay data shown in this study). Furthermore, our immunoblotting analysis showed that, in the absence of inducer, OsTIR1-F74A expressed from the strong *Padh1* promoter did not obviously alter the protein levels of Mcm4-3×sAID and Sec8-3×sAID (Figure  6). Thus, under the experimental conditions we have used, basal degradation is not an apparent problem with our improved AID system for fission yeast. If this problem arises under other experimental conditions, the myriad of solutions mentioned above can be applied to address it.

The strengths and advantages of *S. pombe* as an experimental model organism rely on the continuing development of technologies empowering its use in biological research. Understanding gene functions is a core pursuit of biological research. Our improved AID system is a new addition to a long list of tools and methods for creating conditional loss-of-function mutants in *S. pombe* (Basi *et al.* 1993; Rajagopalan *et al.* 2004; Boe *et al.* 2008; Kearsey and Gregan 2009; Kanke *et al.* 2011; Tang *et al.* 2011; Pai *et al.* 2012; Watson *et al.* 2013; Ding *et al.* 2014; Watson *et al.* 2021). This system will facilitate the analysis of gene functions in *S. pombe*, especially the functions of essential genes.

## Data availability

The RNA-seq sequencing reads were aligned to the *S. pombe* reference genome sequence using HISAT2 version 2.1.0 with the options “–min-intronlen 28 –max-intronlen 820” (Kim *et al.* 2019). The CDS coordinates of protein-coding genes were from the annotation file Schizosaccharomyces_pombe.ASM294v2.44.gtf (ftp://ftp.ensemblgenomes.org/pub/fungi/release-44/gtf/schizosaccharomyces_pombe/, last accessed November 19, 2021). Reads mapped to each CDS were counted by featureCounts version 2.0.1 with the options “-p -s 0 -F SAF” (Liao *et al.* 2014). Differential expression analysis was performed using the R package DESeq2 version 1.28.1 (Love *et al.* 2014). Volcano plots were generated using the R package EnhancedVolcano version 1.6.0 (https://github.com/kevinblighe/EnhancedVolcano, last accessed November 19, 2021). Sequencing data have been deposited at NCBI SRA under the BioProject ID PRJNA743700. The accession numbers of the data of the control samples are SRR15041253 and SRR15041254. The accession numbers of the data of the 5-adamantyl-IAA-treated samples are SRR15041255 and SRR15041256. The accession numbers of the data of the NAA-treated samples are SRR15041257 and SRR15041258. Supplementary material is provided at figshare: https://doi.org/10.25387/g3.16837087.
